# Advances in Electrochemical Nano-Biosensors for Biomedical and Environmental Applications: From Current Work to Future Perspectives

**DOI:** 10.3390/s22197539

**Published:** 2022-10-05

**Authors:** Rabeay Y. A. Hassan

**Affiliations:** 1 Applied Organic Chemistry Department, National Research Centre Dokki, Cairo 12622, Egypt; ryounes@zewailcity.edu.eg; Tel.: +20-11292-16152; 2 Nanoscience Program, University of Science and Technology (UST), Zewail City of Science and Technology, Giza 12578, Egypt

**Keywords:** biosensors, nanomaterials, environmental detection, biomedical diagnosis, wearable devices, machine learning, artificial intelligence (AI)

## Abstract

Modern life quality is strongly supported by the advances made in biosensors, which has been attributed to their crucial and viable contribution in point-of-care (POC) technology developments. POC devices are exploited for the fast tracing of disease progression, rapid analysis of water, and food quality assessment. Blood glucose meters, home pregnancy strips, and COVID-19 rapid tests all represent common examples of successful biosensors. Biosensors can provide great specificity due to the incorporation of selective bio-recognition elements and portability at significantly reduced costs. Electrochemical biosensor platforms are one of the most advantageous of these platforms because they offer many merits, such as being cheap, selective, specific, rapid, and portable. Furthermore, they can be incorporated into smartphones and various analytical approaches in order to increase their sensitivity and many other properties. As a very broad and interdisciplinary area of research and development, biosensors include all disciplines and backgrounds from materials science, chemistry, physics, medicine, microbiology/biology, and engineering. Accordingly, in this state-of-the-art article, historical background alongside the long journey of biosensing construction and development, starting from the Clark oxygen electrode until reaching highly advanced wearable stretchable biosensing devices, are discussed. Consequently, selected examples among the miscellaneous applications of nanobiosensors (such as microbial detection, cancer diagnosis, toxicity analysis, food quality-control assurance, point of care, and health prognosis) are described. Eventually, future perspectives for intelligent biosensor commercialization and exploitation in real-life that is going to be supported by machine learning and artificial intelligence (AI) are stated.

## 1. Fundamentals of Biosensors

In the past decades, the needs for developing chemical sensors and biosensors have rapidly increased due to severe environmental and health challenges. Conventional methods for chemical analysis offer many advantages, including their high accuracy and acceptable sensitivity. However, those analytical methods, in many cases, require complex instruments, high expensive reagents, large sample consumption, and lack of portability and cannot support on-site monitoring, and a lab specialist with high skills is required [1]. Hence, there is a need for developing chemical sensors and biosensors. Accordingly, Clark and Lyons have started the fast development of biosensors and their related aspects directly after introducing the primary glucose oxidase biosensor that was invented in 1962. Since then, many interesting sensor and biosensor applications have been described, and some of them have been commercialized. The simplest way to define a biosensor is: “an analytical device which includes a biologically active element or component in a close contact with an appropriate physicochemical transducer to generate measurable signal (optical, electrical, or electrochemical) directly proportional to the concentration of target substance(s)” [2].

**Figure 1:**
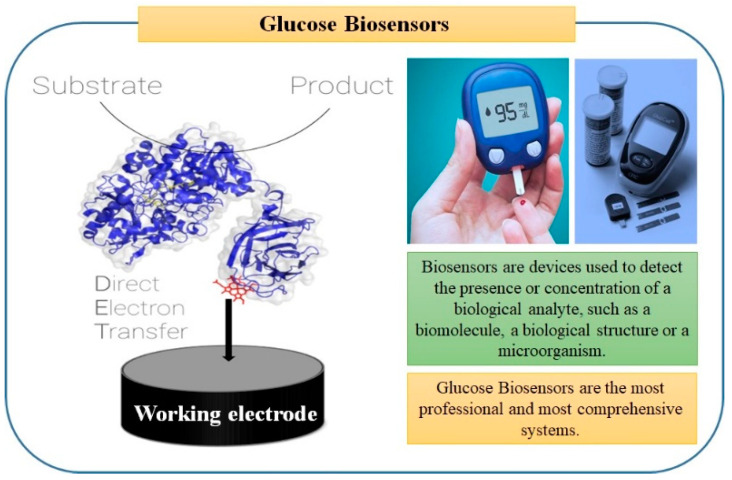
Glucose biosensors, the most common example of electrochemical biosensors, consists of a digital pocket (portable reader) and a sensor chip (a three-electrode setup represents a reference electrode, a counter, and a working electrode). The selective determination of glucose concentration in samples is catalyzed by the enzymatic function of glucose oxidase loaded onto the surface of the working surface [10,11].

Thus, biosensors are bendable detection techniques that have high importance, being able to resolve a potential number of problems and challenges in diverse areas like homeland safety, drugs and pharmaceutical analysis, environmental monitoring and food safety, explosives, and defense-related issues [3,4]. In terms of sampling conditions, biosensors can be directly used to examine the target analytes in a variety of complex samples without any prior sample pre-treatments [5]. Besides, the rapid and accurate recognition of simultaneous multi-targets with high selective identification, full-automation, and a reduction of costs and sample size could be obtained [6]. Herein, the most common known example in our daily life is the glucometer (blood glucose monitoring, displayed in Figure 1).

**Figure 2:**
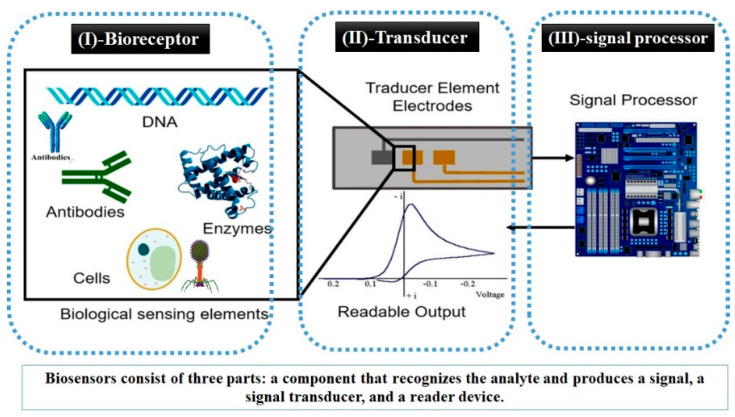
A common diagram displaying the main biosensor’s building blocks including: (**I**) a bio-receptor, (**II**) a transducer, and (**III**) signal processor [12,13].

A biosensor consists of three main constituents: a bio-recognition element (which is also known as a bio-receptor or a bio-sensing element), a transducer, and a signal processor (a schematic diagram of the construction and the main building blocks comprising a typical biosensor is demonstrated in Figure 2). In the first compartment, a bio-receptor is designed and selected for the high specific identification of a selected analyte. Then, the transducer, which is linked directly to the bio-receptor, is converting the biological as well as the biochemical responses into a quantifiable signal. The invisible/small signal inputs from the transducer is eventually magnified to greater output signals that comprises the important waveform features of the input signals. Thus, a digital signal processor collects the magnified signals that can be displayed, analyzed, and saved into the device internal memory [6]. Such electronic components enable detecting, recording, and transmitting the obtained data. The main biosensor advantages (i.e., the high selectivity, as well as the high-sensitivity features) are critically regulated and influenced by the selection of a bio-receptor and a viable method to fix it onto a transducer surface [7]. In terms of performance factors, interaction(s) between the bio-recognition sites and the objective under sensing has to be resistance to the change in the pH of the measurement, temperature, and stirring conditions or to the addition of a foreigner species (i.e., non-targeting analyte). The bio-receptors should be capable of retaining their orientations, structures, functions, and biological activities during biosensor function. As a result, selecting an effective and correct technique for the immobilization of the sensing element is very crucial, and it is one of the keys controlling biosensors performance [8]. Otherwise, a deactivation, misorientation, or leaching out from the surface of the transducer would have occurred. In this regard, two common immobilization techniques are widely used including both chemical (covalent binding) and physical (adsorption or attachment) methods. According to the analyte properties alongside the type of bio-recognition element, the best-fitting immobilization method is then decided [7,9].

The development of novel biosensors is one of the recent trends taking place worldwide. Thus, biosensor research is expected to drive innovations in various fields including point-of-care (POC), wearable, implantable, and miniaturized biosensor devices. Thus, in this review article, the fundamentals and impact of nanomaterials on the structure and design of nanosensors and biosensors were demonstrated. In addition, future perspectives on the strong expected contribution of artificial intelligence (AI) and machine learning in data analysis and visualization applied to biosensing were discussed.

## 2. A Brief History of Biosensors

In the first generation, the biosensor measured the biproduct outcome of the analyte–bio-receptor reaction, which is diffused to the surface of the transducer to generate a quantifiable response. This type of sensor was also known as a mediator-less biosensor. In this regard, the biosensor’s father (Leland Charles Clark) defined the components of a biosensor-setup in his first report that was published in 1956 about the oxygen sensors for blood measurements [14]. In 1962, the first experimental trial for the amperometric detection of glucose using an enzymatic-based electrode was conducted by Clark and collaborators, described in [15]. Then, in 1967, Hicks and Updike modified Clark’s approach by introducing the first glucose oxidase-based electrode, and they implemented this biosensor as an oxygen sensor. Afterwards, in 1969, Guilbault and Montalvo constructed the first enzymatic-based potentiometric biosensor wherein they optimized the potentiometric assay to be applied for enzymatic urea determination [16,17]. Later, in 1973, further modification was carried out by Guilbault and Lubrano, who simultaneously measured both lactate and glucose by the enzymatic-based biosensor relying on the amperometric detection of hydrogen peroxide at a platinum disc-electrode [18]. In this sensor’s construction, the metallic surface of the working electrode was fully covered by a thin film of an immobilized active enzyme in order to provide the high signal stability. Subsequently, particularly in 1974, Klaus Mosbach’s research group created the first ”thermistor biosensor”, which is a heat-sensitive enzyme sensor [19]. Then, in 1975, an optical biosensor was designed by Lubbers and Opitz, who manipulated the concept of the enzymatic biosensor to be applied for the optical determination of alcohol [20]. A series of achievements in biosensor development has been made, and continuous improvement led to the birth of the second generation of biosensors. In the second generation, electron transfer mechanisms from the active center of the immobilized bio-receptors (mainly enzymes) to the conductive electrode surface was the main concern for several research groups. Therefore, individual components, such as artificial redox mediators (electron carriers or electron acceptors in the sensing systems) like menadione, ferricyanide, ferrocene, and *2,6*-dichlorophenolindophenol (DCIP), were integrated to facilitate the electron transfer and to amplify the generated signal [21,22,23]. These new classes of biosensors were called mediator-biosensors, and they led to a definite analytical enhancement in the sensitivity and limit of detection aspects. Technically, the redox mediators could be implemented within the biological matrix (e.g., cross-linked with enzymes, antibodies, or bio-receptors), or they could be added freely (in a soluble form) into the measuring solutions. The first mediated-biosensors was practically introduced in 1976 by Clemens et al. when a “bedside artificial pancreas” was fabricated and provided as an electrochemical glucose biosensor [23]. Later in the same year (1976), another mediated-biosensor approach was reported for blood lactic acid determination using a lactate dehydrogenase-based biosensor. In that study, La Roche manufactured a fully automated lactate-monitor, which was used for mediating the electron transferring from the active metabolic substrate to the electrode surface [24].

The completion of the first and second generation of biosensors has led to exploring a third generation; the cost and feasibility of biosensing and portability were the main driving forces of the third generation of biosensors. Subsequently, Liedberg (1983) implemented the surface plasmon resonance (SPR) technique to define the reactions’ dependency in real-time monitoring [24]. Then Higgins et al. in 1984 invented a pen-sized amperometric detector that was sponsored by Cambridge, USA [25]. A new biosensor generation, the most recent one, was developed through the era of nanotechnology, wherein the powerful characteristics of nanomaterials were exploited in the fabrication of nano-sensors and nano-electronics [26]. Ultimately, from the Clark biosensing approach (the first sensor model) until now, great achievements and huge advancements have been made in biosensor progression.

## 3. Electrochemical-Based Biosensors

Electrochemical sensing and biosensing systems offer the superior capability of multiple analyte(s) detection in complex samples (such as serum and other clinical specimens), with high selectivity and sensitivity. In this sensing platform, bio/electrochemical events (mediated or non-mediated processes) are taking place at the interface of electrochemical transducer surface (working electrode surface). In addition, the selective binding affinity, alongside the catalytic activity between an analyte and a fixed or immobilized bio-receptor could be screened and determined since the generated electrochemical signals could be recorded. Potentiometric, amperometric, conductometric, impedimetric, and voltammetric techniques are the main electrochemical methods used for constructing, adjusting, and optimizing tremendous electrochemical sensors and biosensors [27]. A synopsis of each of these electrochemical techniques will be pointed out in further subsections.

### 3.1. Potentiometric Biosensors

A potentiometric biosensor is defined as an electrochemical device that integrates a biological sensing element with an electrochemical transducer (the working electrode) to generate a difference in the electrical potential [28]. To convert a certain biochemical activity into an electric potential signal, ion-sensitive field-effect transistors, or specific ion electrodes (which are also the so-called ion-selective electrodes (ISEs)) could be applied. Using a selective bio-receptor (e.g., peptides, aptamers, antibodies, or enzymes), adaptable potentiometric sensing protocols could be designed for a wide range of different target analytes. Recently, potentiometric nano-biosensors were developed for the rapid diagnosis of COVID-19 using a three-dimensional molecular imprinted polymer (MIP) [29]. Reflecting the high selectivity of this COVID-19-potentiometric biosensor, discrimination between the targeting coronavirus and other viruses including influenza virus (H1N1 and H3N2) and Middle-East respiratory syndrome (MERS) was achieved. Besides, the virus biosensors reached a very low limit of detection for both spike proteins and a pure suspension of viral particles, with 100 pg/mL and 200 PFU/mL, respectively.

### 3.2. Amperometric Biosensors

A three-electrode setup or two-electrode configuration could be applied for operating an amperometric technique. At a fixed voltage, referring to a reference electrode (e.g., Ag/AgCl or calomel), the sensor drives a faradaic current occasioned by any redox reaction(s) that might occur at the surface of the working electrode. The value of the generated redox current is dependent on the concentration of the analyte that is presented in a supporting electrolyte [30]. The working electrode could be made of or modified with nanomaterials such as carbon-based materials (e.g., graphite, graphene, or carbon nanotubes), noble metals (e.g., gold, platinum, copper), or metal oxide (indium tin oxide (ITO). Amperometric sensing techniques offer great advantages including a wide dynamic–linear response, high precision, fast reading, and high sensitivity. These listed features acquired by this electrochemical method qualified it for mass production and commercialization. Nevertheless, the weakness of selectivity represented by the high interferences and cross-reactivity with other electroactive substances is considered a huge disadvantage and drawback [31]. Recently, an amperometric biosensor was developed by Yaping Dong et. al. and used for the clinical evaluation of creatinine in renal function evaluation. As shown by Yaping’s group, the sensors exhibited promising applicability for on-site medical examination, as well as at-home testing [32].

### 3.3. Conductometric Biosensors

Conductometric biosensors can detect any electrochemical reactive change occuring in a solution. Thus, any change in the ionic composition of the tested sample due to chemical and biochemical reactions taking place could be determined [33]. The conductometric biosensors exhibited several advantages: (I) they do not need a reference electrode; (II) a thin-film electrode is appropriate for miniaturization and large-scale production using inexpensive materials; (III) conductometric transducers are not light-sensitive, and their power consumption is significantly low. Conductometric biosensors were developed using a thin-layer of Au, Cr, Cu, and Ni, which formed on the electrode surface. These modified electrodes were exploited for the immobilization of two active enzymes (glucose oxidase and urease) that were applied for the bio-catalytic oxidation of glucose and urea on the electrode surface [34].

### 3.4. Impedimetric Biosensors

**Figure 3:**
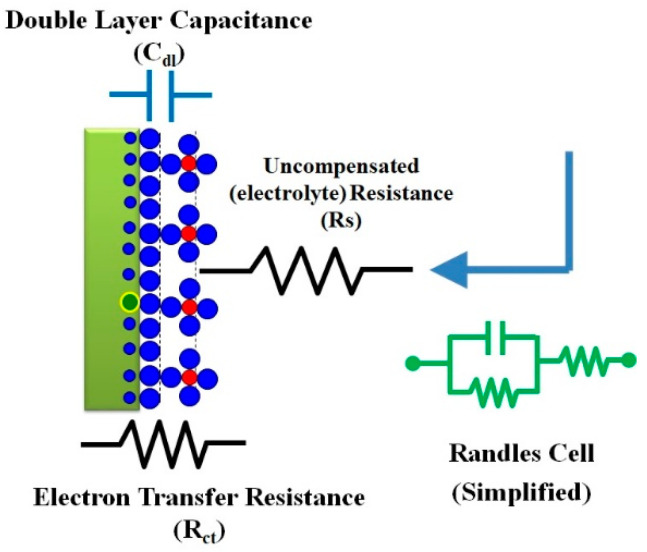
A simple demonstration to visualize what is going in the EIS systems during the conducting of experiments and before generating the EIS signals. At the electrode surface (definitely the side that faces the electrolyte containing the electroactive redox probe), oxidation and/or reduction will take place, which leads to an electron transfer or electron exchange. The diffusion, as well as the catalytic properties, will create different resistances including the charge transfer resistance (R_ct_). To manipulate the EIS date, simulation or modeling through drawing on an electrical circuit (Randes cell) made of double-layer capacitors, solution resistance, and charge transfer resistance is needed.

**Figure 4:**
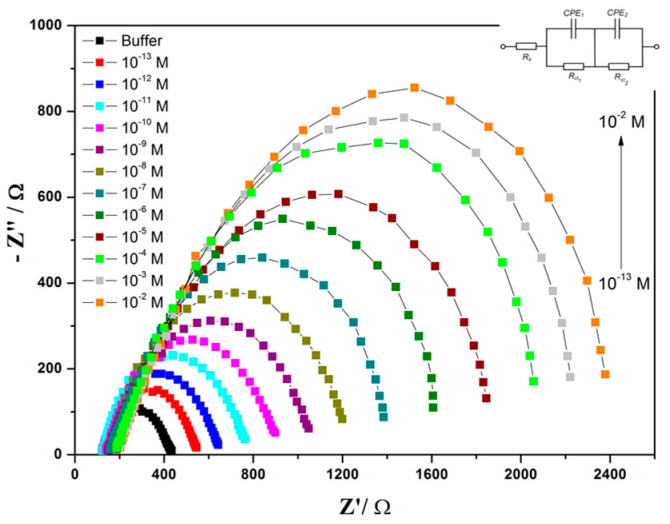
Impedance data in the Nyquist plot format showing up the change of the charge transfer resistances (R_ct_) due to the addition of a different concentration of cadmium ions into the electrochemical cell, which is connected with a disposable screen-printed electrode modified with a ligand/nanocellulose nanocomposite. (The equivalent circuit, inserted figure, was used for quantitative analysis [44].

Impedimetric biosensors, the most powerful electrochemical biosensing technique, record the electrical impedance created at the interface of a solid electrode surface when a small AC potential is applied, then the changes in resistances are measured as a function of frequency [35]. A simple demonstration to visualize what is going on in the EIS systems during the conducting of experiments and before generating the EIS signals is shown in Figure 3. The EIS representation is expressed as an imaginary impedance (Z_imag_) plotted on the *Y*-axis, and a real impedance (Z_real_) plotted on the *X*-axis to form the Nyquist plot, an example of which is shown in Figure 4. Each point on the Nyquist [lot is the impedance at one frequency point, while the Z_imag_ (the imaginary impedance) is expressed in negative values. On the *X*-axis, the right side of the plot is positioned at the low frequency data and higher frequencies are allocated on the left. Among the electrochemical biosensors, EIS is a very sensitive technique for the investigation of interfacial properties that could be conducted at the functionalized surfaces. Therefore, bio-recognition events such as the antigen–antibody capture, protein–protein interaction, drug–target interaction, or whole microbial cell activity could be kinetically monitored. Several impedimetric biosensors were designed using antibodies or aptamers to fabricate impedimetric-immunosensor or impedimetric-aptasensors, respectively [36,37]. In the impedimetric-immunosensor, antigen–antibody specific immune-interaction(s) enable the formation of an immune-complex at the sensor surface that is the point of EIS measurements. On the other hand, the impedimetric-aptasensor represents the immobilization of short single-stranded oligonucleotides, which are so-called aptamers (e.g., RNA or DNA) with high stability and strong binding affinity [38]. Because of that, electron transfer/charge transfer resistance increases. Thus, the EIS-biosensors enable the label-free detection of biomolecular-recognition actions [39]. The use of EIS systems in biomedical and environmental analysis is increasing due to their readiness for lab-on-a-chip fabrication with facile manipulation and the capability to conduct onsite determination [40,41]. On the other hand, EIS is a non-destructive technique that is not limited to biosensor applications but can be exploited to characterize new materials by providing electrochemical information about the ongoing processes such fuel cell electrochemical performance, the formation or inhibition of corrosion, the charging/discharging of batteries, or any other electrochemical process [42,43].

### 3.5. Voltammetric Biosensors

Voltammetric biosensors analyze the target concentration by determining the generated faradaic current through the variation in the electric potential. The advantages of these sensors include the possibility of measuring multiple analytes (simultaneous analysis) [45]. The voltammetry measures both the variable electrical potential (*X*-axis) and electric current (*Y*-axis). Because of the oxidation or reduction of electroactive analyte at the working electrode surface, the peak position at a certain potential value will be used for identification (analyte character cathodic/anodic peak potentials), while the concentration of the corresponding species is reflected by the intensity of the peak current (Faradic current). Among different types of voltammetric techniques, cyclic voltammetry (CV) is the most common one for obtaining quantitative, as well as qualitative, data. Extensive information on the kinetics of electron transfer, the thermodynamics of redox reactions, and the rate of diffusion, as well as the rate of adsorption processes, could be obtained from the CV data. Various voltammetric biosensors for heavy metals (e.g., Pb(II), Cr(VI) and Cd(II), and Hg(II)) determination in environmental samples [46,47,48,49], glucose and urea monitoring in blood using disposable sensor chips [50,51], pharmaceutical compounds in plasma and dosage forms [52], and pathogen biomarkers in microbial cultures using macromolecules such as crown-ethers [46]) have been reported and discussed.

## 4. Impact of Nanomaterials on Biosensor Performance

To construct an effective and high-performance electrochemical sensor and biosensor, working electrode materials must satisfy certain criteria such as biocompatibility, high electrical conductivity, catalytic activity, eco-friendly, and low cost. Moreover, electrode materials have a significant influence on the thermodynamics, as well as the kinetics redox reactions (e.g the electron transfer taking place at the interfaces), and thus they frequently define and support the success of electrochemical processes.

**Figure 5:**
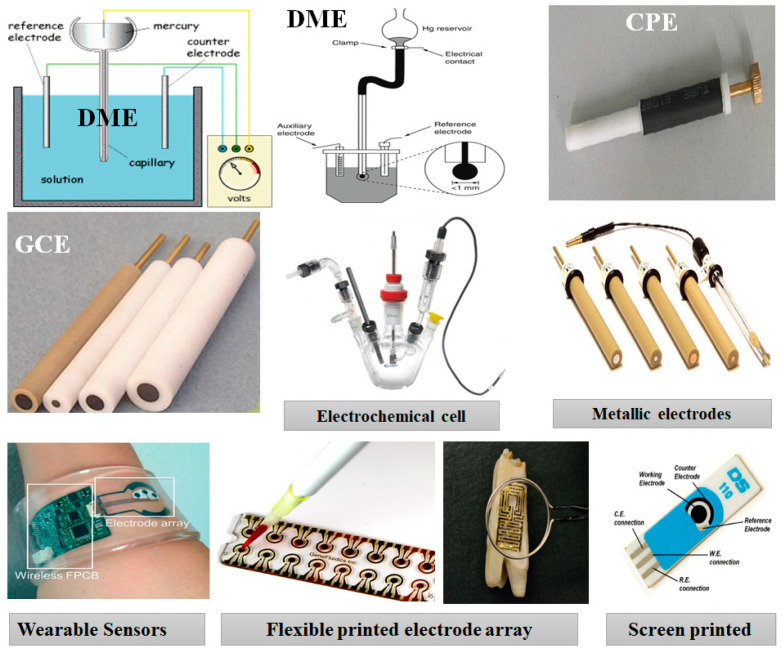
The most commonly used working electrode types and materials in electrochemical sensors and biosensors.

The most commonly used working electrode materials are metallic electrodes (platinum, silver, gold), carbon-based electrodes (e.g., glassy carbon electrode (GCE), carbon paste electrode (CPE) [53], and graphite electrode (GE)). Mercury electrodes (such as a dropping mercury electrode (DME) and a hanging mercury drop electrode (HMDE)) have been conventionally used in polarography [54]. Figure 5 collected the most common working electrodes starting from the DME, ending with the disposable screen-printed electrodes. The toxicity of mercury limited and forbade its uses, while the other electrodes showed some undesired features including their large sizes, which require large electrochemical cells and too much of the analyte, in addition to the difficulty with portability and disposability. Thus, such classical working electrodes are used for the lab investigations and material characterizations.

**Figure 6:**
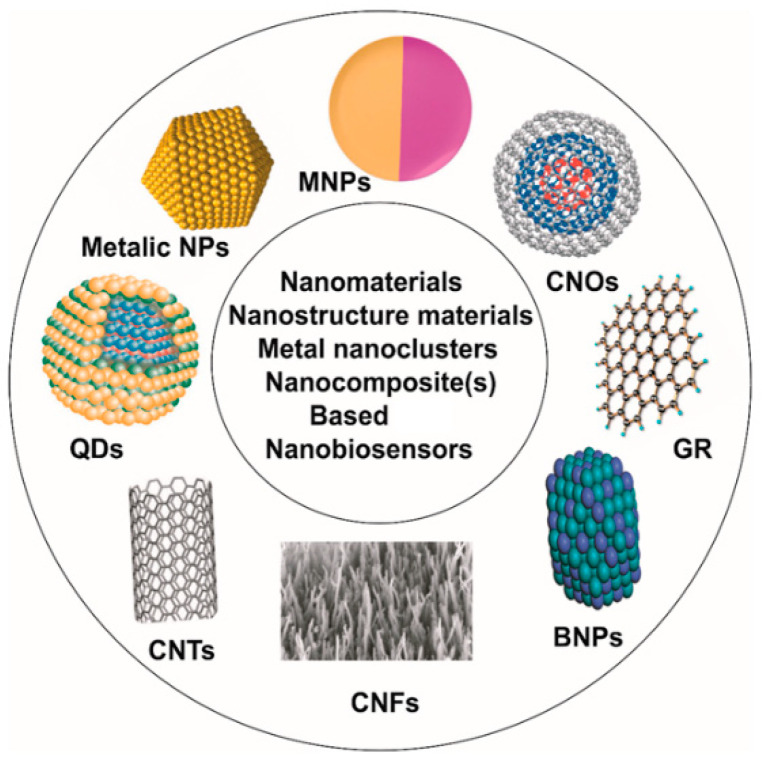
Most commonly used nanomaterials, metal nanoclusters, or nanocomposites for the fabrication of electrochemical nano-biosensors (CNFs: carbon nanofiber, BNPs: bimetallic nanoparticles, GR: graphene, CNOs: carbon nano-onions, MNPs: magnetic nanoparticles, QDs: quantum dots, CNTs: carbon nanotubes, and NPs: nanoparticles). This Figure is adapted from Rizwan and Mohd-Naim et al. [68].

Nanomaterials (synthesis and applications) are strongly involved in tremendous applications including biomaterials, catalysis, nanobiosensors, and nano-bioelectronics [55]. The use of nanoparticles, nanotubes, nanowires, nano-pores, or any other nanostructures in biosensors and the fabrication of devices for diagnosis have been intensively explored [56,57]. Nanomaterials are used for the manufacturing of electrochemical transducers or the functionalization of solid surfaces. The engineered nanostructured materials, with a desirable bio-compatibility, offer an expandable surface-to-volume ratio due to their nanoscale size, and they provide high mechanical strength, electrical conductivity, and catalytic activity [58]. Therefore, they are used often to enhance biosensor performance, amplifying the electrochemical readouts, increasing the sensitivity, and providing a low limit of detection of several orders of magnitudes. In terms of the molecular orientation and structure stability of the immobilized bio-recognition elements, nanostructured-sensor platforms are excellent substrates to efficiently maintain the alignments and orientation while sustaining the bioactivity of the immobilized biomolecules. Moreover, direct charge/electron transfer is facilitated when certain nanomaterials are implemented [59]. For example, carbon nanomaterials have been utilized for the conjugation of biomolecules (DNA, antibody, enzyme, or whole cells) with electrochemical transducers [60]. According to their dimensions and sizes, nanomaterials are classified into four types, including zero dimension, one dimension, two dimensions, and three dimensions. In zero dimensions (0D), metallic nanoparticles (e.g., silver, gold, platinum, gold, and palladium) and quantum dots have all their three dimensions of materials exist in nanoscale. Nanoparticles can be spherical in size, with an average diameter of 1–50 nm. In one dimension (1D), only one dimension is in the range of 1–100 nm, and the other two dimensions can be in macroscale. Examples of 1D nanomaterials include nanowires, nanofibers, nanorods, and nanotubes. In two dimensions (2D), two dimensions are in nanoscale, and one dimension is in macroscale. Coating solid surfaces with nanomaterials creates 2D-like structures (e.g., nano-sheets, nano-walls, nano-thin-films, or nano-thin-multilayers) [61]. Keeping the thickness constant in the nanoscale range, the total area of nanomaterials’ 2D-like structures could be within some square micrometers. Regarding the 3D nanostructures, all dimensions are in the macro-scales, and there are no dimensions in nanoscales. Thus, the 3D nanostructures are bulk materials that contain individual components (blocks) in nanoscale (less than 100 nm). Optical and electrochemical nano-sensors are the most widely used detection modalities due to their simple operation and portability [62]. Examples of nanomaterials that have been frequently exploited in the fabrication and functionalization of nano-biosensors is denominated in Figure 6. A wide range of nanostructured materials has been extensively used as sensing platforms with an expandable surface area for better receptor–analyte interaction and for enhancing electrochemical signal outputs. Carbon nanomaterials, including carbon nanofibers, carbon nanotubes, graphene, or fullerene (C_60_), are perfect materials for sensor fabrication due to their advantages of high electrical conductivity, large surface area, easy functionalization, and their high biocompatibility. Metal and metal oxides nanostructures are predominant materials used for sensor modification because of their high electrocatalytic activity and their tendency to facilitate the electron transfer/charge directly in mediator-less biosensing systems. Thus, ZnO, CuO, NiO, TiO_2_, and Fe_3_O_4_ have been widely used in electrochemical biosensing to promote faster electron transfer kinetics between the electrode and the active sites of immobilized bio-receptors, which led to synergistic enhancement in sensing performance [13].

Precious metal nanostructures, including Au, Pt, Ag, and Pd, have been exploited for electrode modification due to their inertness against oxidation reactions and good biocompatible properties [63,64,65,66]. These nanostructures could be arranged on the electrode surface, or they could be mixed with other components (e.g., polymeric or sol-gel materials) in the electrode matrix [67]. Ultimately, based on the advantages of those unique properties of nanomaterials, nano-sensor devices allow the fabrication of nano-sensor chips and portable sensing devices for rapid and accurate multi-target in complex biological and environmental matrices.

## 5. Biomedical and Environmental Applications

Electrochemical biosensors are widely exploited in miscellaneous applications such as microbial detection, cancer diagnosis, toxicity analysis, and food quality-control assurance, point of care, and health prognosis. In the next subsections, discussion and sorting examples of some of these applications will be handled.

### 5.1. Nano-Electrochemical Biosensors for Microbial Detection

Microbial infections have dramatically spread over the past few decades to be considered one of the most urgent global challenges. Delaying diagnosis might lead to death [69,70]. Thus, early detection (as much as possible) for virulence factors, pathogens, and biological toxins is very necessary to prevent the progression of infection and to provide effective treatments. Electrochemical immune-sensors are among the most common biosensing platforms for microbial detection and identification [71]. The signal is usually obtained through the immobilization of a bio-recognition element on the working electrode surface. The assay steps often end with the injection of a secondary enzyme-labeled antibody that follows the addition of a proper enzymatic substrate [72]. In addition to immunosensors, several methods were used to enhance the selectivity and sensitivity of the electrochemical biosensors. Molecularly imprinted polymers (MIPs)-based electrochemical biosensors are one of those methods. MIPs were created via chemical, photochemical, or electrochemical methods in the presence of a target template molecule(s). MIPs’ exploitation using completely microbial cells as a template have been reported for many applications, including microbial fuel, specific cell capture, cell sorting, separation, and microbial detection. MIP biosensors were also fabricated for the rapid capture and identification of *Salmonella typhimurium* in minced beef samples [73]. Using cyclic voltammetry, the assay optimization and validation have been achieved, reaching a high selectivity and sensitivity against the imprinted cells of *S.* *typhimurium.* Bacteriophages can serve as high-efficiency bio-receptors for the development of electrochemical biosensing platforms. Bacteriophage-sensing platforms have high specificity against their bacterial host [74,75]. In addition, in terms of thermal stability and sensitivity to the changes in surrounding pHs, or solvents, a bacteriophage-based biosensor is more preferable than the immunosensors [74]. In this regard, Mona Tolba and her team developed an impedimetric phage-based biosensors for the selective determination of *Listeria innocua serovar* [76]. As a biorecognition element for this sensor, the bacteriophage endolysin was chemically conjugated onto the screen-printed electrode surface via classical EDC/NHS chemistry. With their developed biosensors, Tolba’s team reached a limit of detection of 10^5^ CFU and 10^4^ CFU/mL, in 2% milk and in a pure culture, respectively. A collective summary of nano-electrochemical transducers’ involvements in the microbial sensors’ construction is displayed in Table 1**.**

### 5.2. Nano-Electrochemical Biosensors for Toxin Detection

As the microbial extracellular-secreted toxins (e.g., hemolysins, leukotoxins and enterotoxins) are the major causative agents of the poisoning and as these toxins may persist even after disinfection, their presence and biological activity have to be determined [100,101]. In this regard, the electrochemical determination of staphylococcal enterotoxin B (SEB) was achieved when Nodoushan and his team succeeded in developing a nanostructured aptasensor using a screen-printed electrode modified with reduced graphene-oxide–gold nanourchins. Differential pulse voltammetry (DPV) was used in the quantitative analysis of the selected enterotoxin, and a linear range from 5.0 to 500.0 fM was obtained with a calculated limit of detection of 0.21 fM [102]. Another electrochemical immnuo-biosensing approach for the determination of Staphylococcal enterotoxin B (SEB) was presented by Chatrathi and his coworkers [103]. A selective antibody was chemically immobilized onto the gold electrode surface through the chemical cross-linking with the amine- or sulfhydryl-reactive heterobifunctional cross-linker. The sensitivity of this immunosensor reached 1.0 ng/mL that corresponds to 5.0 pg in a 5 µL sample. Besides, a nanostructured electrochemical immune-biosensor was developed for the rapid identification of the staphylococcal enterotoxin B. Hence, the chemical conjugation of the selected antibody was conducted on the electrode surface, which is modified with rGO-chitosan-AuNPs. The developed biosensor exhibited high sensing performance due to the flat two-dimensional configuration and large surface area. A limit of detection of 5.0 ng/mL of the targeting SEB was obtained with a sensing time estimated by 35 min [104]. In general, nanomaterial-derived biosensors offer the rapid and sensitive detection of food toxins [105].

On the other hand, neurotoxicity is a major causative factor for Parkinson’s and Alzheimer’s diseases as the common neurodegenerative illnesses [106]. One major group of chemicals with neurotoxic effects is organophosphates (OPs). OPs are very toxic to humans and most animals due to the covalent inhibition of acetylcholinesterase (AChE), a key enzyme for the transmission of nervous signals, responsible for the removal of acetylcholine at the synaptic level [107]. Usually, electrochemical OP biosensors using AChE are based on the catalytic production of thiocholine (TCl), which is electro-active, from the substrate acetylthiocholine (ATCl) [108]. The presence of OPs would result in the inhibition of AChE, reducing thiocholine production, thus decreasing the electrochemical signal. However, new bio-receptors, more sensitive and stable, have been evaluated as an alternative to less-specific and less-stable AChE [109,110].

### 5.3. Nano-Electrochemical Biosensors for Cancer Diagnosis

**Figure 7:**
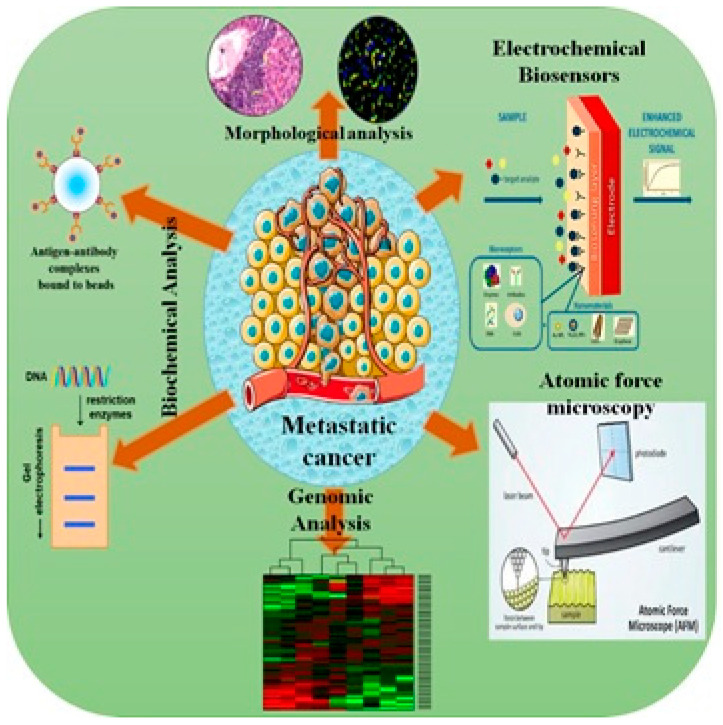
A new vision for cancer investigation through combinatorial biochemical and biophysical techniques.

There are wide varieties of biomarkers (peptides, proteins, antibodies, DNA, RNA, and microRNA) that could be employed as prognostic and predictive tools for the rapid detection of cancer. In the field of cancer biology, anti-proliferative/cytotoxic drug effectiveness and studying various emerging metastatic cancer aspects have been explored using various biosensor techniques [111]. Inspecting specific biological analytes (such as extracellular metabolites, nucleotides, expressed protein biomarkers, or whole cancer cells) is the power to operate electrochemical biosensors. To this end, various types of electrochemical biosensors including microfluidic assays, genosensors, impedance-based biosensors, electrochemluminescence biosensors, and immunosensors were developed. In a recent review article, an interesting integration between the classical cytochemical methods and bioelectrochemical and biophysical techniques (such as electrochemical impedance spectroscopy, cyclic voltammetry, electron microscopy, and atomic force microscopy, as shown in Figure 7) is suggested for the effective understanding of cancer metastasis and apoptosis [111].

Since Caspase-3 (Cas-3) is a crucial mediator in the extrinsic apoptosis signaling cascade, several electrochemical assays were developed for its rapid and sensitive detection [112,113,114]. The cellular microenvironment and cancer cell viability were investigated electrochemically using a 2D- and 3D-sensing cell culture flask integrated with an electrochemical station for recording the obtained electrochemical signals due to metabolic activity and/or extracellular changes. Nano-fabricated sensor chips are fixed on standard cell culture flasks to allow the electrochemical inspection of the cell activity during the cultivation and growth cycles [115]. Those systems have been applied for measuring the electrochemical behaviors of breast cancer cells, as well as brain tumor cells. Furthermore, cancer cellular respiration was measured using the amperometric oxygen sensor, whereas different incubation conditions were applied [116]. Moreover, electrodeposited iridium oxide films were implemented for sensing cellular acidification using a potentiometric pH sensor. The designed biosensing setup enabled an initiative for pursuing 3D-cell biosensor-based cell cultures [117]. An aptasensor was designed for the impedimetric biosensong of Cyt-c using silver nanocluster conjugation [118]. In another cancer biosensor report, an immunosensor was developed for the direct recognition of Bcl-2 and Bax in cancer cell cultures [119]. Further analysis for cancer biomarkers considered one of the anti-apoptotic biomarkers, named Survivin (Sur) [120], whereas a microchannel cyto-sensor was designed to selectively capture it using the anti-survivin oligonucleotide sensor [120]. Besides, a trans-membrane protein known as epithelial cell adhesion molecule (EpCAM) was electrochemically determined using an impedimetric immuno-based biosensor [121]. A colon-cancer-secreted biomarker (IL-13Rα2) has amperometrically been detected [122], whereas its in situ expression was analyzed either in the intact colon cancer or in the lysed cells. In another study, an aptasensor was constructed to recognize L-tryptophan (Trp) based on constant current–potentiometric striping analysis [123]. This Trp-aptasensor proved to be sensitive, with a limit of detection of 6.4 ×10^−11^ M. In another biosensor report, the metastasis of pancreatic tumor cells has been monitored via biosensing the expression of trypsin in cell lysate, which changes its level in cases of pancreatic cancer [124]. In a recent study, simultaneous analysis for metastatic biomarkers programmed death ligand-1 (PD-L-1) and hypoxia-inducible factor-1 alpha (HIF-1α) was developed and offered a detection limit value of 86 pg/mL [125].

To summarize the roles of electrochemical biosensors in cancer metastasis and apoptosis detection, the simple design of biosensing devices make them a reliable tool for the early detection of specific biological targets by converting a biological entity (cell viability, protein binding affinity, and DNA or RNA sensing) into an electrochemical signal that can be measured and analyzed. Besides, cancer biosensors have the ability to determine the real-time continuous responses of cancer or normal cells to discover potential anticancer agents. The future directions for cancer electrochemical sensors are directed towards the development of new functional polymeric substrates (biocompatible and flexible chips) for effective cell adhesion and proliferation or nanostructured biomaterials to be valid for nano-fabrication techniques.

### 5.4. Nano-Electrochemical Biosensors for Viral Infection Diagnosis

Biosensors have generated a great interest in monitoring infectious diseases, especially those caused by microbial pathogens, such as fungi, bacteria, and viruses. Molecularly imprinted polymers (MIPs) are reported to have promising biological applications due to their intrinsic binding affinity to specific proteins and other biological materials. Numerous researches addressed the use of MIPs for accurate viral diagnosis in real-time [126]. MIPs fabricated by Piletska et al. were applied for virus identification and the screening of different epitopes of Adeno-associated viruses (AAV) immobilized on glass beads using polymeric nanoparticles of acrylate and methacrylate [127]. On the other hand, a biosensor was fabricated for the viral hexon protein (the most accessible and abundant surface protein of the human adenovirus type 5 (hAdV5) icosahedral capsid). This biosensor was applied as the template molecule for the entire virus recognition. To validate the sensor’s selectivity, two different viruses, including hAdV5 and minute virus of mice (MVM) were exposed to the sensors with no obvious binding affinity [128].

The virus-neutralizing capacity of hydrogel-based MIPs were produced using a model mammalian virus (porcine reproductive and respiratory syndrome virus (PRRSV-1)) [129]. During the optimization procedure of this biosensor, specificity of virus neutralization, alongside the influence of incubation time on sensor performance, were studied. The sensor’s selectivity was assessed by comparing their neutralizing effects on PRRSV-1 to the effects on the unrelated bovine viral diarrhea virus-1. As a result, no significant cross-reactivity was detected. Thus, the MIPs demonstrated effective virus neutralization in just 2.5 min, and their effect was concentration-dependent. An impedimetric MIP-based sensor was constructed for the detection of dengue virus in the early stage of the infection in serum samples exploiting the self-polymerization of dopamine in the presence of target non-structural protein 1 (NS1), whereas the detection limit was 0.3 ng/mL [130]. A magnetic molecularly imprinted resonance light-scattering sensor was also created for the detection of Japanese encephalitis virus (JEV) within 20 min based on the polymerization of tetraethyl orthosilicate (TEOS), whereas a linear dynamic range of 0.02–2.0 nM was achieved with a limit of detection of 0.1 pM. The sensor’s recovery was evaluated to range from 88% to 107%, to support the determination of the virus in human serum samples [131]. Antibodies-conjugated polyaniline nanowires embedded in graphene quantum dots and nano-gold were used for the detection of hepatitis E virus (HEV) in clinical samples. Introducing an external electrical pulse during the HEV accumulation step enhanced the sensor sensitivity against the targeting virus due to the expansion of the sensor’s surface and the expanding antibody-conjugated polyaniline chain length. The sensor was then used to monitor various HEV genotypes, including G1, G3, G7, and ferret HEV obtained from a cell culture supernatant and in a series of fecal specimen samples collected from G7 HEV-infected monkeys [132]. Zika virus (ZIKV) rapid detection and identification were carried out by the development of electrochemical biosensors based on surface-imprinted polymers and graphene oxide composites. As a result of electrical signal changes with changing virus concentrations, virus quantitative measurements were achieved [133]. Foot and mouth disease (FMDV) virus diagnosis was carried out using viral imprinted polymer (VIP)-based electrochemical biosensors [134]. Two different serotypes (FMDV-O and FMDV-SAT2) have been identified and determined in real animal samples using two different voltammetric VIP biosensors. In terms of serotype O diagnosis, selective bio-recognition components were formed on a gold screen-printed electrode (SPE) via the electrochemical polymerization of the oxidized form, O-aminophenol (O-AP), with the in-activated whole-virus particles. CV, atomic force microscopy (AFM), field emission-scanning electron microscopy (FE-SEM), and Fourier-transform infrared spectroscopy (FTIR) have been used for the VIP surface characterizations. A cross-reactivity study was carried out on several interfering viruses including FMD serotypes A, SAT2, and lumpy skin disease virus (LSDV). With high selectivity, a limit of detection and quantification of 2 ng/mL and 6 ng/mL were obtained, respectively [135]. On the other hand, another VIP biosensor was designed for the FMDV–SAT2 serotype through the direct electrochemical deposition of FMDV inactivated particles within the poly(o-phenylenediamine) (PoD) film on gold–copper nanostructured electrodes. This VIP biosensor was used for the determination of SAT2 serotype in real clinical field specimens without sample treatment [134]. MIP-based electrochemical sensors were developed using poly-m-phenylenediamine on gold electrodes for the SARS-CoV-2-N-protein. The resulting limits of detection and quantification were 15 and 50 fM, respectively [136]. Lately, a double-mediated impedimetric virus biosensor was designed for the rapid detection of the whole SARS-CoV-2 particles. In this study, a mixture of lipophilic electron shuttle (DCIP) and hydrophilic one ferricyanide (FCN) was used for enhancing the electrochemical signals. Additionally, a nanocomposite (carbon nanotubes/tungsten oxides) was exploited for enlarging the imprinted surface area. The sensor provided a very rapid and on-site investigation of whole-virus particles in clinical specimens directly [137]. For ensuring high selectivity, several respiratory interferent viruses were tested, including influenza A viruses (H1N1, H5N1, and H3N2), influenza B, human coronaviruses (hCoVs)-OC43, NL63, 229E, and Middle-East respiratory syndrome coronavirus (MERS-CoV).

Recently, a FET-based biosensor device has been built by immobilizing SARS-CoV-2-S-protein-specific antibodies on graphene nano-sheets. The device can detect the S-protein in the range from 1 to 100 fg/mL. Further, a gold-nanoparticle-based surface-enhanced Raman scattering (SERS) immunobiosensor has been established for the detection of SARS-CoV-2-S-protein in a detection range of 0.77 to 6.07 fg/mL in the PBS and untreated saliva [138]. Besides dual-labeled antibodies tethered to magnetic nanobeads, the sensor has been integrated into the microfluidic chip to detect SARS-CoV-2 nucleocapsid protein in serum at concentrations of 10 to 50 pg/mL in diluted and whole serum. The chips were applied as a smartphone-based diagnostic as well [139]. In conclusion, virus biosensors represent effective diagnostic methods for rapid response and are highly sensitive, with accurate selectivity and the quantitation of viruses in real samples without labeling and without the need for the extraction or purification of genetic material (DNA and RNA) biological molecules, such as DNA or RNA.

### 5.5. Nano-Electrochemical Biosensors for Heavy Metal Detection

The electrochemical sensing of a variety of environmental targets, such as heavy metals, is strongly dependent on the material types and material structures of the working electrodes. Thus, the surface modification of working electrodes can be further improved for high-specific-recognition elements and high selectivity towards different heavy metal ions. The development of an electrochemical setup using electrodeposited platinum nanoparticles on a glassy carbon electrode (Pt NPs/GCE) for the direct detection of arsenic ions (As^3+^) using cyclic voltammetry has been achieved by Dai et al. The voltammetric method was optimized to offer a low limit of detection (35 µg/L) [140]. In another study, a polymeric nanocomposite consisted of polyurethane doped with platinum nanoparticles (PU/Pt NPs) with an average size of 2–5 nm was chemically synthesized and applied as a sensing platform for the direct voltammetric detection of Cu^2+^. A linear range was obtained from 100 to 1000 ng/mL and the limit of detection was 16.72 ng/mL with no interference from different ions. The applicability of this sensor was studied using serum, urine, and acidified tap water samples [141].

Potentially hazardous levels of hexavalent chromium(VI) have been determined in environmental samples using gold-nanoparticle-based electrochemical sensors. The electrochemical parameters have been idealized, and the assay ultrasensitive detection limit was reached (2.38 ng/L) [142]. In addition, one of the most toxic elements (mercury(II)) was also determined electrochemically using nanomaterial-based electrodes. In this regard, poly(ester-urethane) urea conjugated with gold nanoparticles (PUU/Au NPs) was used to modify a carbon paste electrode (CPE) to implement the highly sensitive detection of mercury ions in fish tissue [143]. The sensor showed a linear range of 5 to 155 ng/mL, while the calculated values of the limit of detection and limit of quantification were 0.235 ng/mL and 0.710 ng/mL, respectively. One of the carbon-based nanomaterials, carbon nanofibers (CNFs) were synthesized and characterized to be applied in the electrochemical determination of lead ions (Pb(II). This study was demonstrated by Robinson et al. [144], wherein nano-electrode arrays were fabricated from the vertically aligned forms of carbon nanofibers (VACNFs). The nano-electrodes enhanced the anodic stripping voltammetric performance for the lead ion determination, while the limit of detection was found to be 1.73 nM.

Furthermore, the sensitive and selective impedimetric detection of cadmium ions (cadmium (II)) was achieved when disposable screen-printed electrodes were modified with a nanocellulose/ligand/CNT/Co_3_O_4_-nanocomposite.

Thus, the nanocellulose-intercalated nanomaterial improved the electrochemical assay, whereas the limit of detection was found to be 1.5 × 10^−13^ M [44]. To conclude the importance of nanomaterials in heavy metal detection using electrochemical sensors, metal nanoparticles and carbon-based materials have been extensively investigated as electrode modifiers and reported for various heavy metal analyses in environmental and biological samples [141,143].

## 6. Global Market of Biosensors

Biosensor demands are expanding and receiving huge attention due to a variety of biomedical applications, a growing diabetic population, a high need for portable diagnostic devices, and quick technological advancements. Accordingly, accurate and early disease diagnosis is critical for a positive disease prognosis and patient survival. The demand for disposable, wearable, user-friendly, and cost-effective devices with quick response times has increased rapidly in recent years. These devices have made rapid progress in the medical field due to their ability to meet these criteria through an interdisciplinary combination of approaches from chemistry, biology, virology, nanotechnology, medical science, and electronics. Nano-biosensors, which incorporate nanotechnology, are expected to find beneficial uses in various industrial actions, such as food quality control monitoring, imaging operations, and pathogenic activity detection. Hence, the biosensors market is consolidating due to the rising popularity of medical equipment and tailored medications, the increased preference for disposable and non-invasive biosensors, and increased research collaboration and agreements between diverse manufacturers and research institutions. The forecast of the global biosensors market size was valued at 24.9 billion US dollars in 2022 and is expected to expand at a compound annual growth rate (CAGR) of 8.0% from 2022 to 2030. Biosensors have great abilities in the diagnosis of infection and biomarker diseases. Thus, plenty of biosensing technologies will be available in the market. Electrochemical and optical biosensors are the most used techniques for the development of point-of-care devices (POC) for the quantitative determination of biomarkers, as well as for infectious diseases.

## 7. Conclusions, Future Remarks, and Perspectives

As an effective analytical tool, biosensors have been involved in important fields, such as biomedical diagnostics, disease monitoring, and environmental analysis. A biosensor is a system that can offer a quantitative and selective tracking of a single- or multiple-targeting analyte(s), e.g., cancer biomarkers, DNAs, toxins, heavy metals, drugs, toxic gases, etc.), exploiting the existence of one or several bio-recognition elements and a signal transduction compartment. Among the common biosensors, electrochemical biosensors are the most extensively investigated types, as they offer the advantage of a low detection limit, high specificity, simplicity of construction, and ease of operation. As a new biosensor generation, nano-electrochemical sensors and nano-biosensors have been developed using versatile nanostructures, which led to great enhancement in biosensor performance. Moreover, and because of all the progress and advancement made for electronic instrumentation, versatile, multiplexed, and cost-effective bio-sensing portable digital devices have been produced and commercialized as lab-on-chip devices for on-site and at-home diagnosis. Accordingly, about two-hundred companies are investing in biosensor and bioelectronic fabrication and commercialization. However, from all existing products, about 85 to 90% are going for glucose monitoring in blood patients. The other biosensing platforms are still considered laboratory-based experiments or laboratory-designed platforms. Thus, to transfer those laboratory-designed biosensing platforms to the diagnostic market (i.e real commercialization), strong cooperation is required within the industry and researchers from universities and academic institutes (covering all disciplines and needed backgrounds from materials science, chemistry, physics, biomedicine, microbiology/biology, and engineering). This academic–industry cooperation is urgently needed in order to solve and overcome the challenges and problems in terms of sensor stability, cost, lifetime, and accuracy and precision. From another perspective, future wearable biosensing devices will enable the non-invasive (no-pain) monitoring and testing for various analytes including glucose, metabolites, proteins, and nucleic acids, eventually permitting sophisticated performance and self-diagnosis. Consequently, wearable biosensing devices are predicted to be the fashions of modern biosensors (starting from wrist-mounted, stretchable, soft chips to fashion accessories and daily textiles). However, medical doctors and biomedical societies still reject and resist using such technology (i.e., non-invasive biosensors) without extensive validation and successful applications in human testing with better understanding of the clinical relevance of sensor information. Towards the far future of nano-biosensors and wearable devices, integration between artificial intelligence (AI), machine learning (ML) methods, nanotechnology, and nano-electronics could achieve impressive advances in designing and fabricating smart nano-biosensors that satisfy the biosensors global market and impact sensor commercialization.

## Figures and Tables

**Table 1:** Examples of microbial detection using nanomaterial-modified electrochemical sensors.

Bacterium(a)	Transducer	Chemistry	Bio-Receptor	LOD	Reference
*E. coli* O157:H7	Gold	EDC/NHS	Antibody	2 CFU/mL	[77]
*E. coli* O157:H7	Nanoporous aluminum oxide membrane	Trimethoxysilane-HA-EDC/NHS	Antibody	10 CFU/mL	[78]
*E. coli* O157:H7	Nanoporous aluminum oxide membrane	Silane-PEG	Antibody	10 CFU/mL	[79]
*E. coli* K-12	Gold microelectrode, interdigitated	Physisorption	T4 bacteriophage	10^4^–10^7^ CFU/mL	[80]
*E. coli* K-12	Boron-doped microelectrode array	Physisorption	Antibody	NA	[81]
*E. coli* O157:H7	Gold microelectrode, interdigitated	Physisorption	Antibody	2.5 × 10^4^ CFU/mL	[82]
*E. coli*	Gold	SAM-EDC/NHS	Antibody	1.0–10^3^ CFU/mL	[83]
*E. coli*	Gold electrode	SAM-biotin-NeutrAvidin	Biotinyl antibody	10 CFU/mL	[84]
*E. coli*	gold-tungsten plate wire	Polyethyleneamine-streptavidin	Biotinyl antibody	10^3^–10^8^ CFU/mL	[85]
*E. coli*	Gold disk	SAM	Synthetic glycan	10^2^–10^3^ CFU/mL	[86]
*E. coli*	Polysilicon interdigitated electrodes	Glutaraldehyde	Antibody	3 × 10^2^ CFU/mL	[87]
*E. coli* O157:H7	Gold	SAM-HA-EDC/NHS	Antibody	7 CFU/mL	[88]
*E. coli*	Gold	SAM-PDICT cross-linker	Bacteriophage	8 × 10^2^ CFU/mL	[89]
*E. coli*	Graphene paper	Biotin-streptavidin	Antibody	1.5 × 10^2^ CFU/mL	[90]
*E. coli*	Screen-printed carbon microarrays	EDC/NHS	Bacteriophage	10^4^ CFU/mL	[91]
Sulfate-reducing bacteria	Glassy carbon	Reduced graphene sheet with chitosan–glutaraldehyde	Antibody	1.8 × 10^1^ CFU/mL	[92]
Sulfate-reducing bacteria	ITO	Chitosan-reduced grapheme sheet	Bioimprint of bacteria	1.0 × 10^4^ CFU/mL	[93]
Sulfate-reducing bacteria	Ni-foam	Nanoparticle-SAM-EDC/NHS	Antibody	2.1 × 10 CFU/mL	[94]
*Salmonella typhimurium*	Gold	SAM-glutaraldehyde	Antibody	NA	[95]
*Salmonella typhimurium*	Electroplated gold	MHDA-EDC-NHS	Monoclonal antibody	10 CFU/100 mL	[96]
*Salmonella typhimurium*	Gold	Polytyramine-glutaraldehyde	Antibody	NA	[97]
*Campylobacter jejuni*	Glassy carbon	Physisorped onto *O*carboxymethylchitos-modified Fe_3_O_4_ nanoparticles	Monoclonal antibody	1.0 × 10^3^ CFU/mL	[98]
*Listeria innocua*	Gold	SAM-EDC/NHS	Endolysin (bacteriophage-encoded peptidoglycan hydrolases)	1.1 × 10^4^ CFU/mL	[76]
*Staphylococcus aureus*	Nanoporous alumina	Silane-GPMS	Antibody	10^2^ CFU/mL	[99]
*Porphyromonas gingivalis, E. coli*	Microfluidic cell	Impedance reading during flow of cells	None	10^3^ cells/mL	[77]

## Data Availability

Not applicable.
